# The effects of a structured badminton program on social and emotional learning in adolescents

**DOI:** 10.7717/peerj.21808

**Published:** 2026-10-28

**Authors:** Veli Başal, Pablo Prieto-González, Maria Isip

**Affiliations:** 1 Faculty of Sports Sciences, Aksaray University, Aksaray, Turkey; 2 Sport Sciences and Diagnostics Research Group, College of Sciences and Humanities, Prince Sultan University, Riyadh, Saudi Arabia

**Keywords:** Social learning, Emotional learning, Badminton, Adolescent

## Abstract

**Background:**

This study aimed to examine the effects of a structured badminton exercise program, which naturally incorporates social interaction, decision-making, and emotional regulation elements inherent to the sport, on the social and emotional learning (SEL) levels of adolescent students.

**Method:**

A quasi-experimental design with a pretest–posttest control group was employed. The study included a total of 65 adolescent students (32 females and 33 males; mean age = 12.98) attending a public middle school in Aksaray Province. A structured badminton exercise program was implemented for the experimental group for 12 weeks, twice a week during physical education classes, with each session lasting 90 minutes. No additional intervention was applied to the control group, which continued with standard physical education classes. Data were collected using a personal information form and the Social and Emotional Learning Scale for Adolescents (SELS). A 2 × 2 × 2 mixed-design analysis of variance (ANOVA) was conducted to examine changes in SEL scores before and after the intervention and to determine differences according to gender. Effect sizes were calculated using partial eta-squared (*ηp*^2^).

**Results:**

The findings indicated that the experimental group showed a statistically significant increase in post-test SEL scores compared to the control group. No statistically significant main effect of gender was found. However, the mean SEL scores of female students were higher than those of male students, and a significant Time × Group × Gender interaction indicated that the magnitude of improvement was greater among female students.

**Conclusion:**

The findings suggest that participation in a structured badminton program was associated with positive changes in the social and emotional learning levels of adolescent students. No separate SEL curriculum was implemented; the observed improvements emerged naturally as a byproduct of the structured badminton intervention.

## Introduction

Socialization and emotional maturation are critical processes in child and adolescent development and play a decisive role in individuals’ healthy adaptation to society (Gander & Gardiner, 2010; Handel, 2011). Social and emotional learning (SEL) is a holistic process that encompasses individuals’ abilities to recognize and manage emotions, set positive goals, demonstrate empathy, establish healthy relationships, and make responsible decisions (Wigelsworth et al., 2022). SEL programs include practices aimed at supporting individuals’ social and emotional development, prosocial behaviors, interpersonal relationships, and academic achievement (Durlak et al., 2015; Jones & Bouffard, 2012).

One of the primary responsibilities of schools is to create learning environments that support SEL. Such environments not only strengthen students’ abilities to understand emotions, build healthy relationships, and develop positive behaviors, but also contribute to academic achievement, cooperation, and moral character development (Greenberg et al., 2017; Johnson & Johnson, 2009; Yeager, 2017). Adolescence is a critical developmental stage characterized by physical changes, intensified peer relationships, identity exploration, and significant emotional fluctuations (Moffitt et al., 2011; Odak et al., 2023).

Sport activities are considered an effective pedagogical tool for promoting SEL among adolescents. In particular, physical activity-based interventions contribute to the development of skills such as emotional regulation, empathy, social awareness, and responsible decision-making in authentic contexts (Bailey et al., 2013; Eime et al., 2013; Lubans, Plotnikoff & Lubans, 2012). In this context, Bandura’s social cognitive theory and Ryan and Deci’s self-determination theory provide important theoretical foundations. According to social cognitive theory, individuals acquire social and emotional skills through observation, modeling, and reciprocal interaction, while sport environments provide rich contexts for such learning to occur (Bandura, 1986). Self-determination theory emphasizes that sports participation enhances intrinsic motivation and self-regulation by satisfying the needs for competence, autonomy, and relatedness (Ryan & Deci, 2024). Although self-determination theory was originally formulated to explain the sustained, autonomous engagement of individuals in sport over time, its core constructs intersect meaningfully with SEL: the satisfaction of autonomy and relatedness needs through sport participation parallels SEL’s emphasis on self-management and relationship skills, while competence-supportive sport environments are conducive to the self-awareness and responsible decision-making competencies central to the collaborative for academic, social, and emotional learning (CASEL) framework (CASEL, 2021). Taken together, social cognitive theory and self-determination theory complement one another in explaining how structured sport participation can plausibly give rise to SEL-related competencies, with the former emphasizing observational and interactional learning processes and the latter emphasizing the motivational conditions that sustain engagement in those processes.

Badminton, the focus of this study, offers unique advantages among racket sports. As one of the fastest racket sports in the world, badminton requires rapid reaction, instantaneous decision-making, cognitive flexibility, and adaptation to constantly changing game conditions. These characteristics directly support core components of SEL such as self-awareness, self-management, and responsible decision-making. In addition, doubles play and interactions during training facilitate the development of empathy, communication, and social awareness skills through the natural structure of the game (Chen et al., 2022). Recent research indicates that badminton training positively influences emotional regulation and social interaction skills in adolescents (Yang & Chen, 2025; Ying, 2024). These findings suggest that badminton, through both individual and paired game dynamics, may holistically enhance both the emotional and social dimensions of SEL.

It is important to emphasize that the present study did not implement any separate psychoeducational SEL curriculum or explicit social-emotional lessons. The intervention consisted exclusively of a structured badminton training program. Any development in SEL competencies was expected to occur organically as a natural byproduct of the sport’s inherent structure, including paired drills, doubles play, competitive situations, immediate feedback after errors, cooperation, rule adherence, and emotional control during play.

While team sports (*e.g.*, soccer, basketball, or volleyball) strongly contribute to cooperation and collective empathy development, badminton offers a different mechanism through individual responsibility, rapid decision-making, and direct interaction with an opponent. This distinction makes badminton particularly valuable for fostering emotional development and socialization skills. Moreover, its low cost, accessibility, and minimal space requirements provide practical advantages for school-based interventions (Komari et al., 2023; Wu & Li, 2022).

A substantial body of research across disciplines consistently supports the positive effects of sport-based interventions on SEL outcomes. Research indicates that the positive impacts of sport environments on personal, social, and physical development stem from a supportive social climate and a structured, explicit pedagogical approach, rather than occurring automatically (Holt et al., 2017; Opstoel et al., 2020).

Studies in educational games, basketball, and volleyball have similarly demonstrated that sport participation meaningfully contributes to students’ social and emotional skill development (Gould, Martin & Walker, 2022; Hromek & Roffey, 2009; Son & Berdychevsky, 2024). However, the great majority of this evidence concerns team sports or general physical activity programs, and badminton-specific evidence remains confined largely to adult or experienced-athlete samples examining psychological skills, emotional self-regulation, and social development rather than adolescents’ SEL as defined by the CASEL framework (Bebetsos & Antoniou, 2003; Kumari & John, 2023; Wu & Li, 2022). However, there is still a limited number of studies in the literature examining the effects of badminton on adolescents’ SEL through structured interventions. This gap is notable given that, unlike team sports, badminton combines rapid individual decision-making with direct dyadic interaction, a combination whose relevance to SEL development in early adolescence has not yet been empirically tested through a structured, school-based intervention. In particular, research focusing on the role of gender remains scarce (İlhan et al., 2017; Zeze & Erel, 2021). In this regard, Wood & Eagly’s (2012) biosocial construction theory provides an explanatory framework. According to this theory, gender roles are shaped through the interaction of biological, cultural, and social factors, which in turn differentiate the ways individuals express their social and emotional skills. Therefore, it is expected that the effects of sport interventions on SEL may vary by gender, making the inclusion of gender as a variable theoretically necessary (Wood & Eagly, 2012).

Although badminton participation may contribute to a broad range of developmental outcomes, including cognitive functioning, sport-specific skill acquisition, enjoyment, and future intentions to participate in physical activity, the present study was intentionally limited to the examination of SEL. SEL represents a distinct developmental construct encompassing self-awareness, self-management, social awareness, relationship skills, and responsible decision-making. Therefore, while cognitive, behavioral, and sport-specific outcomes are undoubtedly important dimensions of adolescent development, they were beyond the scope of the current investigation. Future studies are encouraged to adopt multidimensional evaluation frameworks that simultaneously assess cognitive, motor, emotional, and motivational outcomes associated with badminton participation.

This study aims to examine the effects of a 12-week structured badminton exercise program on the SEL levels of adolescents aged 12–14 years. Additionally, it investigates whether these effects differ according to gender. The findings are expected to contribute uniquely to the literature on the integration of badminton into school-based SEL interventions and to provide guidance for future research.

## Materials & Methods

### Study design

In this study, a quasi-experimental pretest–posttest control group design was adopted in order to rigorously examine the effects of a 12-week structured badminton exercise program on adolescents’ SEL levels. The implementation process was conducted in a public middle school located in the city center of Aksaray, which reflects the socioeconomic characteristics of the region. Two classes with similar demographic characteristics (age, gender distribution, and academic level) were selected by the school administration in consultation with the researcher. To preserve the natural structure of the school environment and avoid disrupting the ongoing educational process, a quasi-experimental approach was employed in assigning participants to groups, taking into account existing class structures and logistical constraints (Büyüköztürk et al., 2020). The selection of classes was based on the following criteria: similar class sizes, comparable average academic achievement, no prior badminton experience among students, and willingness of the physical education teacher to participate in the study. Accordingly, one class with similar demographic characteristics was assigned as the experimental group and another as the control group; pretest and posttest measurements were completed for both groups without any missing data. The selection of the study group was based on strict criteria, including being between 12 and 14 years of age, having no prior badminton training, and voluntary participation.

Although random assignment of individual students was not possible due to school scheduling and ethical considerations regarding disruption of intact classes, no statistically significant baseline differences between groups were detected (see Participants section). Conducting the study within a single school provided a strategic advantage for enhancing internal validity by ensuring that external variables—such as school climate, physical resources, administrative approach, and environmental factors—were held constant across both groups. Potential interactions between groups were closely monitored by the researcher, and necessary precautions were taken to preserve the integrity of the intervention program. While this single-site approach enabled the intervention to be implemented with high fidelity in a standardized setting, the findings offer an applicable model for adolescent populations in similar educational ecosystems. Although future research is encouraged to include large-scale studies across different types of schools, the homogeneous structure of the present design provided a solid basis for clearly observing the psychosocial effects of badminton.

### Participants

The study included a total of 65 adolescent students (32 females, 33 males) with a mean age of 12.98 years (age range: 12–14), attending a public middle school located in the city center of Aksaray, Türkiye during the 2024–2025 academic year. The experimental group consisted of 34 students (16 females, 18 males), while the control group included 31 students (16 females, 15 males) (Table 1).

Participants were selected using homogeneous purposive sampling, a non-probability sampling method, to ensure that all students had no prior badminton experience and were at an early adolescence stage considered most appropriate for SEL development. The inclusion criteria were: (a) being between 12 and 14 years of age, (b) voluntary participation, (c) written informed consent provided by a parent or legal guardian, and (d) no physical or health condition that would prevent regular participation in physical activity. The exclusion criteria were: (a) prior experience in badminton, (b) the presence of chronic health conditions that could affect participation continuity, and (c) unwillingness to participate in the study.

All participants in the experimental group completed the 12-week program with a high adherence rate (mean attendance: 96.5%). Four students missed a maximum of two sessions due to illness or personal reasons; however, they completed make-up sessions. No participant withdrew from the study during the research process. Baseline comparisons indicated no statistically significant difference between the experimental and control groups in SEL scores prior to the intervention (*p* > 0.05). Equivalence between groups was also examined in terms of demographic variables; no statistically significant differences were found in gender distribution (*χ*^2^(1)=0.19, *p* = 0.66) or age distribution (*χ*^2^(2)=0.08, *p* = 0.96). In addition, both groups were located in similar socioeconomic regions, and none of the participants had prior badminton experience. These findings indicate that no statistically significant differences between groups were detected at baseline in terms of either SEL levels or key demographic characteristics prior to the intervention.

### Measures

Data were collected using two instruments: a personal information form and the Social and Emotional Learning Scale for Adolescents (SELS). The authors received permission to use the SELS from the copyright holder (Totan, 2018).

#### Personal information form

The personal information form, which included students’ gender and age data, was presented on the same page as the Social and Emotional Development Scale.

#### Social and Emotional Learning Scale (SELS)

The Social and Emotional Learning Scale (SELS) was developed based on the theoretical framework of CASEL (2021) to assess adolescents’ SEL levels. The scale consists of 23 items in total. The SELS is rated on a 5-point Likert-type scale ranging from “Not at all true” (1 point) to “Completely true” (5 points). Scoring is performed by summing the item scores directly, and the total score is obtained from the sum of all items. Higher scores indicate a higher level of competence in the respective SEL domain. In addition, a 5-item short form of the scale is available for rapid screening purposes.

**Table 1:** The demographic characteristics of the participants.

**Variable**	**Group**	**Control Group**		**Experimental Group**	
		*n*	(%)	*n*	(%)
Age	12	8	25.8	9	26.5
	13	16	51.7	16	47.0
	14	7	22.5	9	26.5
Gender	Female	16	51.7	16	47.1
	Male	15	48.3	18	52.9

In the original validation study, Cronbach’s alpha internal consistency coefficients were reported to range between .70 and .83 for the subscales and .92 for the total score. In the present study, reliability was recalculated, and the Cronbach’s alpha coefficient for the total scale score was found to be .93. All item-total correlations were positive and above .30, and item discrimination indices were found to be statistically significant in upper–lower group comparisons (*p* < 0.05). The construct validity of the scale was examined in the original development study using confirmatory factor analysis (CFA), which indicated acceptable fit indices for the five-factor structure in an adolescent sample (comparative fit index (CFI) = 0.93, root mean square error of approximation (RMSEA) = 0.06). Since the scale was originally developed for Turkish-speaking adolescents, no translation or cultural adaptation process was required.

In conclusion, the SELS is a valid and reliable measurement tool that enables the individual assessment of children’s and adolescents’ SEL levels and is widely used to evaluate the effectiveness of psychoeducational programs (Totan, 2018). The primary outcome measure in this study was the total SEL score, as it represents a comprehensive indicator of adolescents’ social and emotional competencies aligned with the CASEL framework. Although the scale includes five subscales, separate pre-post analyses for each subscale were not conducted in the present study. This approach was chosen to maintain statistical power with the available sample size and to focus on the overall effectiveness of the intervention, which is the main research aim. Subscale-level explorations are recommended for future studies with larger samples.

### Procedure

This study was conducted in accordance with the ethical principles of the Declaration of Helsinki developed by the World Medical Association. Ethical approval was obtained from the Human Research Ethics Committee of Aksaray University (Protocol No: 2024/01-30, Date: February 28, 2024). Written informed consent was obtained from the legal guardians of all participants, and voluntary assent was also obtained from the students themselves. Participant confidentiality and anonymity were ensured throughout the study, and voluntary participation was upheld as a fundamental principle of the research process.

Pretest data were collected at the beginning of the 2024–2025 academic year through the administration of the Social and Emotional Learning Scale in a classroom setting using a paper-and-pencil format for both the experimental and control groups. The administration of the scale took approximately 10–15 min. Following the pretest, a 12-week structured badminton exercise program was implemented for the experimental group. This program consisted exclusively of badminton-specific technical, tactical, and game-based activities. No separate psychoeducational SEL curriculum or explicit social-emotional lessons were implemented at any stage of the intervention. The program was conducted by the first author, who has extensive experience in physical education and sports, holds a Level 2 badminton coaching certificate issued by the Turkish Badminton Federation, and has 12 years of coaching experience.

The structured badminton program followed a consistent weekly schedule. Each 90-minute session was divided as follows: 15 min warm-up (general mobility and basic footwork), 50 min technical and tactical skill development (racket grip, shuttle control, serve, clear, drop, smash, drive, footwork drills, and paired exercises), 15 min game-based activities (singles and doubles matches with emphasis on fair play and communication), and 10 min cool-down and reflection. The program progressed from basic skills in the first four weeks to more complex game situations in the later weeks. SEL competencies (such as communication, cooperation, emotional regulation, empathy, and responsible decision-making) were expected to develop organically as a natural byproduct of the sport’s inherent structure, including paired drills, doubles play, competitive rallies, immediate feedback after errors, rule adherence, and interactions with partners and opponents.

For full transparency and reproducibility of the intervention, the weekly technical and tactical content and the SEL competency domain most directly supported by the activities are described below. In Week 1, the program introduced racket grip, shuttle control through bouncing exercises, and clear shots, while paired practice provided opportunities to support social awareness. Week 2 focused on serving technique and created opportunities for responsible decision-making during rallies. Week 3 involved a review of clear shots and serves, with activities supporting self-awareness during skill performance. Week 4 introduced drop shots together with partner exercises that provided opportunities for developing relationship skills. Week 5 focused on drop shots and smashes, with an increased emphasis on self-management under greater physical and competitive demands. Week 6 introduced smashes and drives, during which leadership roles naturally emerged within small-group activities. Week 7 consisted of a comprehensive review of the previously learned strokes and continued to provide opportunities for social awareness through interactive practice. Week 8 included a review of serving technique combined with individual goal-setting activities. Weeks 9 and 10 focused on competition preparation, emphasizing cooperation and communication among partners and peers. Weeks 11 and 12 consisted of a knockout-style competition format in which previously acquired technical and tactical skills were applied in game contexts. Throughout the program, each 50-minute technical and tactical block began with individual skill practice and subsequently progressed to paired and small-group exercises. The final 15 min of each session were allocated to singles and doubles games, providing structured opportunities for peer interaction, communication, cooperation, and decision-making during play. The remaining 15 min of each session were devoted to warm-up activities, and the final 10 min were used for cool-down and reflection, resulting in a standardized 90-minute session structure. Exercise intensity was not monitored using a standardized indicator such as session-RPE or heart-rate zones. Therefore, no predefined physiological intensity range was used as an intervention criterion. Perceived difficulty varied across sessions according to the technical and tactical demands of the activities being performed. Progression from one skill level to the next was determined through the coach’s observation of observable improvements in students’ performance. A progression checklist was used to guide and document this process, and no implementation difficulties requiring modification of the progression procedure were recorded. Students who were unable to attend a scheduled session received the content of the missed week individually during a subsequent make-up session. The make-up session focused on the same technical and tactical content that had been missed, allowing the student to complete the planned learning sequence before continuing with the subsequent intervention content.

Each session was conducted according to a pre-prepared lesson plan. Sessions included warm-up activities, badminton skill development drills, partner or small-group practices, game-based activities, and cool-down phases. To ensure implementation fidelity, content alignment, time allocation, and participation levels were recorded by the researcher at the end of each session.

No additional intervention was applied to the control group within the scope of the study. During the research period, the control group continued with standard physical education classes consisting of a variety of general physical activities (*e.g.*, team games such as soccer and basketball, basic fitness exercises, and other sports) aimed at promoting overall physical development. These standard physical education classes were not structured to include badminton-focused activities or any explicit SEL components. Thus, while both groups received equivalent total physical education time (180 min per week), the experimental group’s program differed by its structured and progressive badminton-specific content and the natural SEL opportunities arising from the technical, tactical, interactive, and decision-making demands of the sport.

Posttest data were collected after the completion of the 12-week intervention using the same measurement tool and identical administration procedures as the pretest. Both pretest and posttest administrations were conducted under similar conditions for both groups. The resulting data were analyzed to evaluate the effects of the structured badminton exercise program on adolescents’ SEL levels.

Within the scope of the study, both the experimental and control groups participated in a total of 180 min of physical education per week, consisting of two compulsory physical education lessons (45 ×2=90 min) and two elective physical education lessons (45 ×2=90 min). The badminton program implemented in the experimental group was conducted within these class hours, and students did not participate in any additional training or supplementary physical activity programs. Therefore, there was no difference between the experimental and control groups in terms of total physical activity duration.

Figure 1 was created by the authors and does not contain copyrighted material. The purpose of this program was to examine whether participation in a structured badminton exercise program influences social and emotional learning. Although no explicit SEL activities or psychoeducational components were designed, the social and emotional competencies described in the CASEL (2021) framework were expected to emerge naturally through participation in badminton activities such as partner drills, doubles play, communication, cooperation, and adherence to game rules. Collaborative for Academic, Social, and Emotional Learning is a leading global organization that supports the development of students’ social and emotional learning competencies.

**Figure 1:**
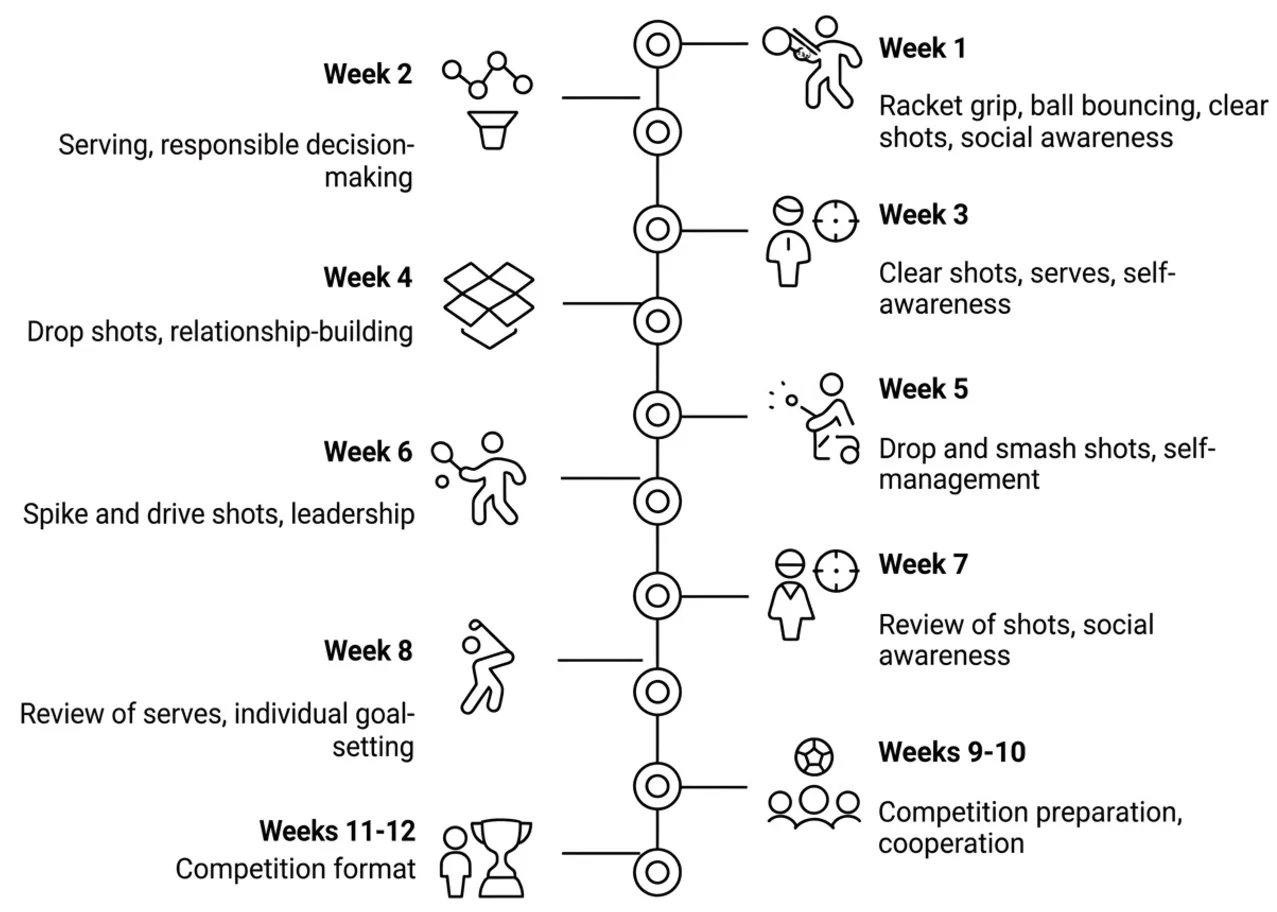
12-week progression of the program, outlining the badminton skills and social-emotional development outcomes addressed each week. The program progresses from fundamental skills, such as grip, serves, and shots, toward self-awareness, self-management, leadership, cooperation, and competition preparation.

### Statistical analysis

Data analysis was conducted using IBM SPSS Statistics 27.0, and the significance level was set at *p* < 0.05. A priori power analysis was conducted using G*Power 3.1 prior to data collection. Assuming a medium effect size (*f* = 0.25), an alpha level of 0.05, and a statistical power of 0.80, the required minimum sample size for a 2 ×2 mixed-design ANOVA was calculated to be approximately 52 participants. The final sample size of 65 participants exceeded this threshold, indicating sufficient statistical power for the study. First, the normality of the data distribution was examined using skewness and kurtosis coefficients. The results indicated that the data were normally distributed. Additional assumptions required for mixed-design ANOVA were also tested. Homogeneity of variances between groups was assessed using Levene’s test, which indicated that variances were homogeneous for both pretest (F(1,63)=2.14, *p* = 0.148) and posttest (F(1,63)=1.87, *p* = 0.176) scores.

Sphericity, an assumption related to the repeated-measures factor, was examined using Mauchly’s test; however, since the study included only two time points (pretest and posttest), the sphericity assumption was inherently satisfied, and the test was not required (Mauchly’s test is applicable only when there are three or more repeated measurements).

Within the scope of the research, data were collected from the experimental and control groups at two different time points, before and after the 12-week structured badminton exercise program. A 2 ×2 mixed-design ANOVA was employed to examine changes in students’ SEL levels over time and to investigate differences in SEL scores according to the gender variable.

## Results

A comparison was made between the pre-test and post-test scores of the control and experimental groups (Table 2). Furthermore, the normality of the data was confirmed before the main analyses. Skewness and kurtosis values were found to be within the recommended range of −2 to +2, and visual inspections of the histograms and stem-leaf plots further supported the suitability of the dataset for parametric testing (George & Mallery, 2019). To evaluate the effects of the 12-week intervention, a 2 ×2 mixed-design ANOVA was performed to compare the total pre-test and post-test SEL scores (Table 3). The primary outcome of this study was the total score of the Social and Emotional Learning Scale (SELS), which provides a comprehensive measure of adolescents’ overall SEL competence consistent with the study’s main objective. No separate analyses were performed for the five individual SEL subscales (self-awareness, self-management, social awareness, relationship skills, and responsible decision-making). This decision was made for the following reasons: (1) the study was specifically designed to examine the holistic impact of the structured badminton program on overall SEL rather than domain-specific effects; (2) with a sample size of 65, conducting multiple subscale analyses would have substantially reduced statistical power and increased the risk of Type I error; and (3) the total SEL score has demonstrated high reliability (Cronbach’s *α* =0.93) and is the most commonly used index in similar intervention studies.

No statistically significant difference was observed in the control group after the 12-week period, and the results indicated only a weak effect size. In contrast, a statistically significant increase in SEL scores was found in the experimental group following the intervention (*p* < 0.005). The presence of a significant change only in the experimental group, while SEL scores in the control group remained relatively stable throughout the study period, suggests that participation in the badminton program was associated with these improvements. However, since both groups continued to participate in standard physical education classes, the interpretation of these findings should be made with caution.

**Table 2:** A comparison of the pretest and posttest scores of the control and experimental groups.

**Variable**	**Group**	***n***	**Pre-test**		**Post-test**	
			x¯	SD	x¯	SD
SELS	Control	31	85.12	6.45	87.25	7.02
	Experimental	34	85.91	7.27	103.91	9.19

**Notes.**

SELS Social and Emotional Learning Scale

**Table 3:** 2 ×2 × 2 mixed-design ANOVA results for SEL scores.

**Effect**	**df**	**Mean Square**	**F**	**%95 CI**	***p***	***η*^2^p**
Time	1	3,339.466	298.841	93.64–97.68	<0.001	0.830
Group	1	2,489.931	23.945	92.49–97.45	<0.001	0.282
Gender	1	20.334	0.196	87.67–93.53	0.660	0.003
Time × Group	1	2,084.156	186.506	93.93–99.68	<0.001	0.754
Time × Gender	1	72.573	6.494	92.42–99.63	0.013^*^	0.096
Group × Gender	1	56.544	0.544	82.33–97.31	0.464	0.009
Time × Group × Gender	1	86.071	7.702	81.43–97.61	0.007^*^	0.112

**Notes.**

* *p* < 0.05.

No significant main effect was found for the gender variable; however, the three-way Time×Group×Gender interaction was statistically significant, indicating that the magnitude of the intervention effect differed by gender. On the other hand, the absence of separate analyses according to age groups (12–14 years) limits the evaluation of age as a potential moderating factor, which is acknowledged as a limitation of the study.

Partial eta-squared (*η*p^2^) was used to determine effect sizes. *η*p^2^ is a commonly used measure for interpreting effect sizes in ANOVA analyses; values of 0.01 indicate a small effect, 0.06 a medium effect, and 0.14 or higher a large effect (Cohen, 1988). In this study, since a mixed-design ANOVA was used to examine pretest–posttest changes and interactions between experimental and control groups within the same model, partial eta-squared (*η*p^2^) was selected as the effect size measure in order to evaluate the relative contribution of each main effect and interaction to the dependent variable while excluding error variance.

The observed change represented a large effect size (*η*p^2^=0.754), indicating a statistically large magnitude of change associated with the 12-week badminton program. However, given the relatively small sample size, single-school setting, non-random group assignment, and reliance on self-report measures, this large effect size should be interpreted cautiously and not generalized broadly. The comparison of pretest and posttest mean SEL scores of the study participants is presented in Table 2.

Table 2 presents a comparison of the pretest and posttest scores of the control and experimental groups. The findings indicate a limited increase in the control group from pretest to posttest. In contrast, a statistically significant increase was observed in the experimental group. Additionally, the posttest scores of the experimental group showed a more consistent distribution, whereas the change observed in the control group remained relatively low. These results demonstrate that the experimental process had a more positive effect on the SEL levels of the experimental group, with an increase of 20.95%.

Table 3 presents the results of the 2 × 2 × 2 mixed-design ANOVA examining the effects of time (pre-test–post-test), group (experimental–control), and gender on students’ SEL scores.

The analysis revealed a significant main effect of time, *F* (1, 61)=298.841, *p* < 0.001, *η*p^2^=0.830, indicating a substantial overall increase in SEL scores from pre-test to pos*t*-test. A significant main effect of group was also found, *F* (1, 61)=23.945, *p* < 0.001, *η*p^2^=0.282. In contrast, the main effect of gender was not significant, *F* (1, 61)=0.196, *p* = 0.660, *η*p^2^=0.003.

Regarding the two-way interactions, the Time × Group interaction was significant and large in magnitude, *F* (1, 61)=186.506, *p* < 0.001, *η*p^2^=0.754, indicating that the increase in SEL scores over time was substantially greater in the experimental group than in the control group. The Time × Gender interaction was also significant, *F* (1, 61)=6.494, *p* = 0.013, *η*p^2^=0.096. However, the Group × Gender interaction was not significant, *F* (1, 61)=0.544, *p* = 0.464, *η*p^2^=0.009.

Most importantly, the three-way Time × Group × Gender interaction was statistically significant, *F* (1, 61)=7.702, *p* = 0.007, *η*p^2^=0.112. This finding indicates that the magnitude of the intervention effect differed according to gender. Follow-up simple effects analyses showed that although both female and male students in the experimental group improved significantly, the improvement was greater among female students.

## Discussion

The findings of this study examined the effects of a 12-week structured badminton program—without any additional explicit SEL curriculum on the social and emotional learning levels of students in early adolescence. The results obtained from the quasi-experimental design indicate that the intervention group demonstrated a statistically significant and practically meaningful increase in SEL scores compared to the control group. Since no separate psychoeducational SEL curriculum was implemented at any stage, and all SEL-related competencies emerged naturally as integral parts of the structured badminton training (through paired drills, doubles play, competitive situations, immediate feedback after errors, rule adherence, and partner cooperation), the findings suggest that the structured badminton exercise program was associated with improvements in adolescents’ social and emotional learning levels. It is important to note that these effects occurred without any added explicit SEL instruction, highlighting the potential of badminton’s natural structure when delivered in a structured school-based format.

These findings are consistent with the limited but growing body of literature suggesting that structured badminton programs can positively influence social and emotional learning during adolescence, thereby providing empirical contributions to this field (Wu & Li, 2022; Kumari & John, 2023). The effect size observed in this study, classified as large according to Cohen’s criteria, points to a statistically robust intervention effect; however, given the small sample size, single-school setting, non-random allocation, and reliance on self-report measurement, this magnitude should not be interpreted as a precise or generalizable estimate of the true population effect, and may partly reflect features of the present study design rather than the intervention alone; similarly designed studies have also reported positive effects of sport-based programs on social development and emotional regulation (Shen, Rose & Dyson, 2022; Wu & Li, 2022). Nevertheless, the results suggest that participation in a structured badminton exercise program was associated with improvements in SEL. The inclusion of SEL-supportive elements such as partner work, cooperative game structures, and reciprocal interaction processes occurred naturally within the pedagogical flow of badminton practice. Accordingly, the findings indicate that badminton, when appropriately structured, may function not only as a physical activity but also as a multidimensional learning environment that can support the development of SEL competencies.

The individual and paired structure of badminton provides athletes with a strong sense of personal accountability for their actions. This feature directly aligns with self-awareness and responsible decision-making skills measured in the present study. However, in the absence of a comparative sport condition, this characteristic should not be interpreted as evidence of superiority over team sports. Recent research indicates that team sports primarily enhance psychological resilience through mechanisms of social support, whereas individual and dual sports operate through self-efficacy and self-regulation processes (Wei, Xue & Sun, 2025). These differentiated mechanisms explain why badminton is worth investigating in relation to SEL, but they do not establish the superiority of one approach over another. This issue should be addressed more comprehensively in future studies comparing different sport disciplines.

Although the present study did not directly measure the mechanisms specific to badminton, existing literature suggests that badminton involves cognitive and social demands relevant to SEL development. Previous studies have highlighted that badminton training supports students’ social interaction processes (Ren et al., 2025; Wang et al., 2025; Wu, Lee & Chen, 2024). No separate SEL enhancement program was implemented apart from the badminton intervention in this study. However, the structure of the implemented program inherently incorporated SEL-related components such as cooperation, communication, rotational leadership roles, group interaction, and conflict-resolution processes. The social and emotional demands naturally embedded within badminton are conceptually consistent with the competencies described in the CASEL (2021) framework. It should be acknowledged that these SEL-related components were inferred from the structure of the program and assessed only through the quantitative SELS total score, rather than through direct observation or participant accounts of how cooperation, communication, or conflict resolution were actually experienced during sessions. Future studies incorporating qualitative interviews or focus groups with participants and the instructor would allow for a more concrete and contextualized understanding of how these SEL-related elements manifest during badminton practice, and could complement quantitative findings such as those reported here.

As outlined in the Introduction, the broader sport-SEL literature, including qualitative meta-study evidence on programs of comparable duration (Holt et al., 2017; Opstoel et al., 2020), consistently points to positive associations between structured sport participation and SEL outcomes, and the large effect size observed in the present 12-week program is consistent with this body of evidence. However, badminton-specific evidence has so far been confined largely to experienced athletes or adult samples (Bebetsos & Antoniou, 2003; Komari et al., 2023; Kumari & John, 2023; Wu & Li, 2022), limiting the extent to which prior findings can be generalized to adolescent populations. The present findings, obtained with an adolescent sample through a structured school-based intervention, may be associated with SEL outcomes in this age group, while also confirming the need for further research to clarify the underlying mechanisms.

From a gender perspective, no statistically significant main effect of gender was found; however, female students showed slightly higher mean SEL scores than male students, and the three-way Time × Group × Gender interaction was statistically significant, indicating that female students benefited somewhat more from the intervention than male students. This finding aligns with some studies reporting similar patterns of social and emotional competencies across genders in sport contexts (İlhan et al., 2017). Conversely, other studies have reported higher SEL-related scores among female athletes compared to males (Zeze & Erel, 2021). Wood & Eagly’s (2012) biosocial construction theory provides a useful framework for interpreting these findings, suggesting that gender differences arise from the interaction between biological predispositions and socialization processes. The significant three-way Time × Group × Gender interaction indicates that the badminton-based program was somewhat more responsive to female students’ SEL development, even though both genders showed significant improvement. Recent studies also suggest that while team sports enhance psychological resilience primarily through social support mechanisms, individual sports improve self-efficacy and self-regulation, and these patterns are observed across genders (Wei, Xue & Sun, 2025). The present findings partially align with this general framework, while also indicating that the magnitude of benefit was not entirely gender-independent; further longitudinal and mixed-methods research is needed to examine gender–sport interactions more comprehensively.

Overall, the findings of this study are broadly consistent with a substantial body of literature suggesting that sport-based interventions can support adolescents’ SEL development (Bailey et al., 2013; Eime et al., 2013; Greenberg et al., 2017; Holt et al., 2017; Lubans, Plotnikoff & Lubans, 2012; Opstoel et al., 2020). The contribution of the present study lies in demonstrating that a structured badminton exercise program, without any added explicit SEL curriculum, can positively enhance adolescents’ social and emotional learning. The combination of technical skill development and naturally occurring group interactions during badminton activities created favorable conditions for fostering self-awareness, social awareness, self-management, relationship skills, and responsible decision-making in an authentic learning environment. Considering the high adherence rates and the absence of detected baseline differences between groups, the findings provide valuable preliminary evidence regarding the feasibility and potential effectiveness of this approach. However, the observed SEL gains should be interpreted within the context of the structured badminton intervention through which social and emotional competencies emerged naturally during participation.

It should be noted that the observed SEL gains should be interpreted within the context of this holistic badminton-based intervention. Future research employing dismantling designs (technical badminton training only *versus* badminton with added explicit SEL components) or active control groups would help clarify the active ingredients responsible for the observed effects. Designs incorporating self-reported cognitive antecedents alongside behavioral outcomes, such as the cluster-randomized physical-education-based behavioral skills training program reported by Coimbra et al. (2021) and the goal-setting intervention examined by Gerani et al. (2020), offer useful methodological templates for disentangling the contribution of structured physical education content from that of explicit skill-training components in future badminton-SEL research.

The strengths of this study include the use of a control group with equivalent physical education exposure, documented intervention fidelity, and a focus on early adolescence. The present study has several limitations that should be acknowledged. The study employed a quasi-experimental design without random assignment of participants to groups due to practical and ethical constraints within the school system. Although two classes with similar characteristics were selected and no statistically significant baseline differences were detected, the possibility of selection bias cannot be entirely ruled out.

Additionally, the study was conducted in a single school, which substantially limits the generalizability of the findings to other regions, school types, and socioeconomic contexts. Future research is encouraged to employ multi-school randomized designs. Additionally, the single-region and relatively homogeneous socioeconomic sample may limit generalizability across different cultural contexts, although this is common in school-based research. Replication studies across diverse regions and socioeconomic settings would strengthen external validity. Another important limitation is the absence of an active control group. Because both groups participated in standard physical education classes, which inherently involve movement, cooperation, and self-discipline elements that overlap conceptually with SEL components, the content and structure of the control group’s curriculum were not systematically documented or compared against the experimental program in this study. Consequently, it cannot be ruled out that some of the SEL-relevant experiences captured by the control group’s relatively stable scores were already embedded in standard physical education content. While the control group continued with standard physical education classes, the experimental group received a structured badminton program that involved greater instructor attention and novelty. Future studies should systematically document and compare the curricular content of control-group physical education classes against the experimental intervention to allow firmer conclusions about the badminton-specific contribution to SEL outcomes, as opposed to that of generic physical education exposure. Therefore, it should be considered that the observed improvements may reflect the combined effect of the direct impacts of the badminton program and other supportive elements that emerged during the intervention process. Finally, because the intervention consisted solely of structured badminton training without any explicit SEL curriculum, it is not possible to fully disentangle the effects of the sport itself from the social and emotional learning components naturally embedded within it (*e.g.*, paired drills, communication, cooperation, and emotional regulation during play). Additionally, the analysis was conducted on the total SEL score rather than examining each of the five subscales separately. While this approach aligns with the study’s primary aim of evaluating overall SEL improvement, it limits the ability to identify which specific SEL domains were most responsive to the intervention. Future studies should consider subscale-level analyses to provide more nuanced insights.

## Conclusion

This study provides preliminary evidence suggesting that a structured badminton program, through its inherent technical, cognitive, social, and emotional demands, offers a promising approach to supporting the social and emotional learning (SEL) development of adolescent students. The findings indicate that a 12-week badminton intervention was associated with improvements in SEL competencies during early adolescence, a critical developmental period. As a racket sport requiring rapid decision-making and interpersonal coordination, badminton offers distinctive opportunities for fostering SEL skills beyond general physical activity programs.

Since no separate SEL curriculum or psychoeducational intervention was implemented, the observed improvements are most plausibly associated with participation in the structured badminton exercise program and the social-emotional demands inherently embedded within the sport; however, given the quasi-experimental design without random assignment, this association should not be interpreted as definitive proof of a causal effect. Although no significant main effect of gender was found, the significant three-way Time × Group × Gender interaction indicates that the magnitude of improvement differed by gender, with female students showing somewhat larger gains than male students. This suggests that badminton-based SEL programs can benefit adolescents of both genders, although the relative magnitude of benefit may not be identical across genders, a point that warrants further investigation in future research. However, the quasi-experimental design without random assignment and the sampling of participants from a single geographic region should be considered when interpreting the findings. Given that both groups continued standard physical education classes, the quasi-experimental design, and the reliance on self-report measures, future studies should employ active control conditions to better distinguish badminton-specific SEL gains from the effects of general physical education. In addition, more detailed investigations of age-related differences are recommended. Future research should also involve more diverse samples and include comparative studies across different sports disciplines to further clarify the role of badminton in SEL development.

##  Supplemental Information

10.7717/peerj.21808/supp-1 Supplemental Information 1 Data

## Abbreviations

SEL: Social and Emotional Learning
SELS: Social and Emotional Learning Scale
SELA: Social and Emotional Learning Levels Assessment
SPSS: Statistical Package for the Social Sciences
ANOVA: ANalysis Of Variance
CASEL: Collaborative for Academic, Social, and Emotional Learning
*η*^2^p: Partial eta-squared
SD: Standard Deviation

## Additional Information and Declarations

### Data Availability

The following information was supplied regarding data availability: The raw measurements are available in the Supplemental File.
